# Supplementation of Magnolol Attenuates Skeletal Muscle Atrophy in Bladder Cancer-Bearing Mice Undergoing Chemotherapy via Suppression of FoxO3 Activation and Induction of IGF-1

**DOI:** 10.1371/journal.pone.0143594

**Published:** 2015-11-24

**Authors:** Meng-Chuan Chen, Yen-Lin Chen, Chi-Feng Lee, Chih-Huang Hung, Tz-Chong Chou

**Affiliations:** 1 Graduate Institute of Medical Sciences, National Defense Medical Center, Taipei, Taiwan; 2 Department of Pathology, Cardinal Tien Hospital; School of Medicine, Fu-Jen Catholic University, New Taipei City, Taiwan; 3 Division of Biopharmaceuticals, Institute of Preventive Medicine, National Defense Medical Center, Taipei, Taiwan; 4 Institute of Medical Sciences, Tzu Chi University, Hualien, Taiwan; 5 Department of Biotechnology, Asia University, Taichung, Taiwan; 6 China Medical University Hospital, China Medical University, Taichung, Taiwan; Faculty of Animal Sciences and Food Engineering, University of São Paulo, BRAZIL

## Abstract

Skeletal muscle atrophy, the most prominent phenotypic feature of cancer cachexia, is often observed in cancer patients undergoing chemotherapy. Magnolol (M) extracted from *Magnolia officinalis* exhibits several pharmacological effects including anti-inflammatory and anticancer activities. In this study, we investigated whether magnolol supplementation protects against the development of cachexia symptoms in bladder cancer-bearing mice undergoing chemotherapy. Combined treatment of magnolol with chemotherapeutic drugs, such as gemcitabine and cisplatin (TGCM) or gemcitabine (TGM), markedly attenuates the body weight loss and skeletal muscle atrophy compared with conventional chemotherapy (TGC). The antiatrophic effect of magnolol may be associated with inhibition of myostatin and activin A formation, as well as FoxO3 transcriptional activity resulting from Akt activation, thereby suppressing ubiquitin ligases MuRF-1 and MAFbx/atrogin-1 expression, as well as proteasomal enzyme activity. Notably, magnolol-induced insulin-like growth factor 1 (IGF-1) production and related protein synthesis may also contribute to its protective effects. The decreased food intake, and intestinal injury and dysfunction observed in the mice of TGC group were significantly improved in the TGCM and TGM groups. Moreover, the increased inflammatory responses evidenced by elevation of proinflammatory cytokine formation and NF-κB activation occurred in the atrophying muscle of TGC group were markedly inhibited in mice of combined treatment with magnolol. In summary, these findings support that magnolol is a promising chemopreventive supplement for preventing chemotherapy-induced skeletal muscle atrophy associated with cancer cachexia by suppressing muscle protein degradation, and inflammatory responses, as well as increasing IGF-1-mediated protein synthesis.

## Introduction

Cancer cachexia has been considered a complex metabolic syndrome that is characterized by anorexia, body weight loss, skeletal muscle atrophy, inflammation, and impaired metabolic functions [1]. Cancer cachexia has a high mortality and morbidity and its prevalence is as high as 86% in patients with advanced cancer [2, 3]. The most prominent feature of cancer cachexia is the severe skeletal muscle mass loss that is closely associated with the tumor size, stage, and the type of anticancer drug used. The increased muscle protein degradation and/or decreased protein synthesis are critical factors causing muscle atrophy. The degradation of muscle protein is mainly regulated by the ubiquitin-proteasome system (UPS) that is composed of ubiquitin-activating enzyme (E1), ubiquitin carrier protein (E2), and ubiquitin-conjugating enzymes (E3 or E3 protein ligase) [4]. When the ubiquitin chain is attached to the targeted protein substrate, the complex can be recognized by the 26S proteasome and digested to peptides [5]. The forkhead box O (FoxO) is a key transcription factor accounting for the transcription of muscle-specific E3 ligase, F-box (MAFbx)/atrogin-1, and muscle ring finger 1 (MuRF-1), which are responsible for muscle protein ubiquitination and degradation by the proteasome [6, 7]. Elevated ubiquitinated protein expression and proteasome activity were observed in atrophying muscles [8]. By contrast, mice deficient in either MAFbx or MuRF-1 exhibit more resistance to muscle atrophy [9], suggesting that suppressing UPS activity may be a key target for attenuating muscle wasting. The mechanisms resulting in muscle atrophy associated with cancer cachexia are very complex and multifactorial, and are mediated by the interplay of tumor factors, host factors, and their interactions. It is known that overproduction of myostatin and activins, nuclear factor-κB (NF-κB)-evoked inflammatory responses, and impaired insulin-like growth factor 1 (IGF-1)-dependent protein synthesis are closely related to the pathogenesis of muscle atrophy [10, 11]. Therefore, regulating these muscle atrophy-related pathways may be a potential strategy for alleviating the muscle mass loss associated with cancer cachexia.

Bladder cancer, the most frequently occurring tumor in the urinary system, has a poor prognosis. Clinically, the combined treatment of gemcitabine (G) and cisplatin (C) is a common chemotherapeutic regimen for bladder cancer [12]. However, numerous deleterious effects, such as organ damage and gastrointestinal mucosal injury, have been observed during chemotherapy [13–15], thereby limiting their application. Furthermore, the body weight loss mainly due to muscle atrophy is frequently seen in cancer patients treated with cisplatin [16]. Although several currently available nutritional, metabolic, and pharmacological treatments are used to prevent cancer cachexia, the outcomes remain poor or unsatisfactory. Therefore, developing safer and more effective chemopreventive adjuvants or supplements to attenuate the toxicity and the development of cancer cachexia during chemotherapy is very urgent.

**Fig 1:**
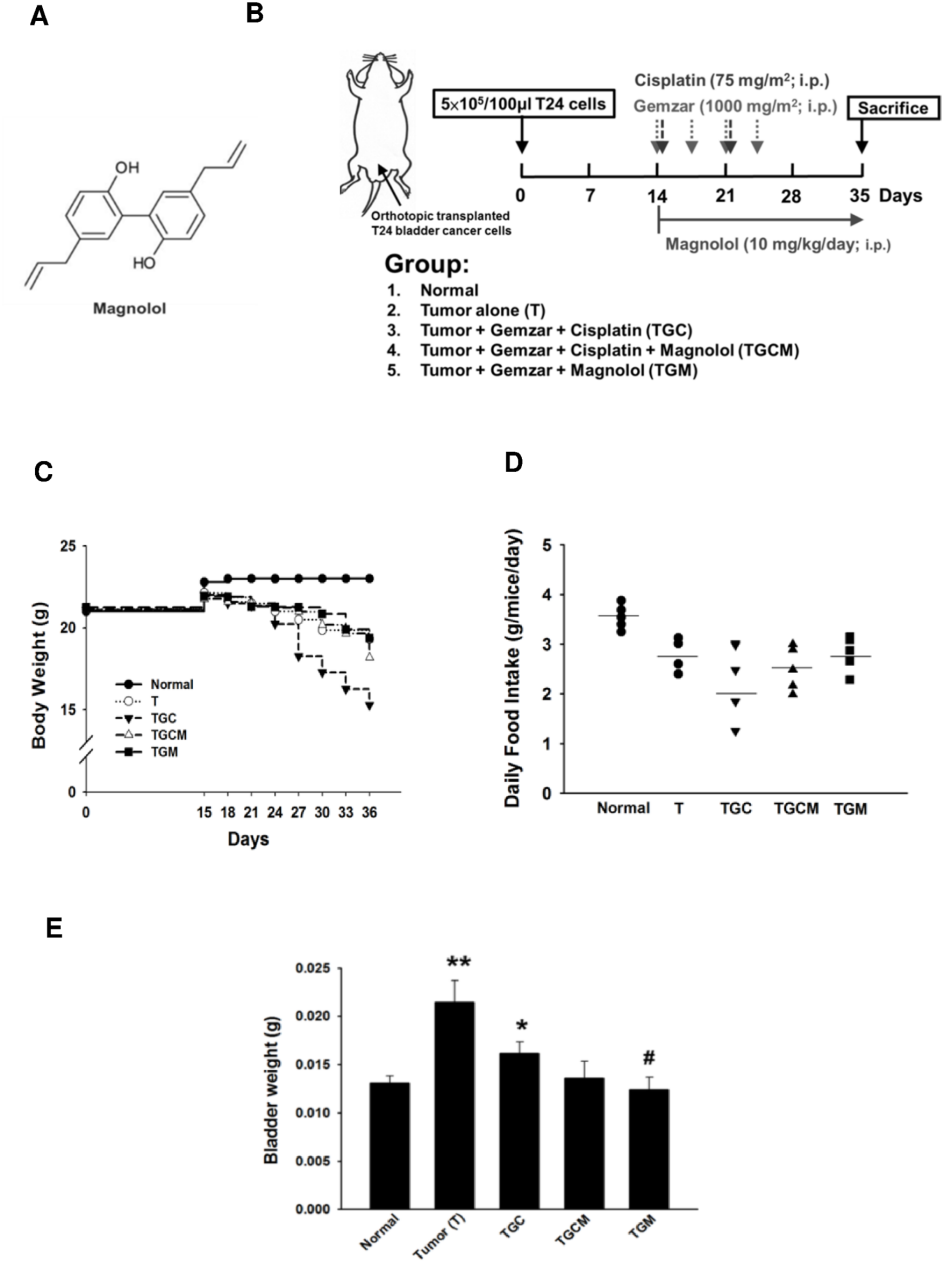
Effects of magnolol on body weight, daily food intake and tumor growth. The chemical structure of magnolol (A) and the experimental design of this study (B) were shown. The body weight (C) daily food intake (D) and bladder weight (E) in different groups were measured. Data was expressed as mean ± SEM (n = 5). *P < 0.05, **P < 0.01 versus normal group. ^#^ *P* < 0.05 versus TGC group.

Magnolol (Fig 1A) isolated from *Magnolia officinalis*, a Chinese herb, possesses several biological functions including inhibition of inflammation, angiogenesis, and cancer growth [17, 18]. However, the effects of magnolol on tumor and chemotherapy-induced cancer cachexia have not been reported. It is the first study to demonstrate that combined treatment with magnolol or replacement of cisplatin with magnolol significantly ameliorate the muscle atrophy in cancer mice undergoing chemotherapy.

## Methods

### Reagents

The T24 human bladder cancer cells were incubated in RPMI 1640 medium containing 10% fetal bovine serum, 2 mmol/L L-glutamine, and 100 U/mL of penicillin—streptomycin. Magnolol with > 98% purity was obtained from the Medical and Pharmaceutical Industry Technology and Development Center (Taipei, Taiwan). Subsequently, magnolol was dissolved in DMSO and diluted as required, and the final DMSO concentration was set at 1% (v/v). The cisplatin and gencitabine were provided by Eli Lilly (Indianapolis, IN, USA). The enzyme-linked immunosorbent assay (ELISA) kits of myostatin, activin A, IGF-1, TNF-α, IL-6, and IL-1β were purchased from R&D Systems, Inc. (MN, USA). Other reagents were purchased from Sigma-Aldrich Corporation (St. Louis, MO, USA). The various antibodies used in the study were shown in Table 1.

**Table 1:** The antibodies used in this study.

Antibodies	Manufactories
TNF-α, IL-1β, IGF-1, MuRF-1, MAFbX-1,β-actin	Santa Cruz Biotechnology (Dallas, TX, USA)
AKT, phospho-AKT, NF-κB, phospho-NF-κB, FoxO3, phospho-FoxO3, mTOR, phospho-mTOR, p70S6K, phospho-p70S6K,4E-BP-1, phospho-4E-BP-1	Cell Signaling Technology (Danvers, MA, USA)
CRP	Novus Biologicals (Littleton, CO, USA)
Myostatin, IL-6	GeneTex, Inc. (Irvine, CA, USA)

TNF-α, Tumor necrosis factor alpha; IL-1β, Interleukin-1 beta; IGF-1, Insulin-like growth factor 1; MuRF-1, Muscle RING-finger protein-1; MAFbX-1, Muscle Atrophy F-Box-1; AKT/PKB, Protein kinase B; NF-κB, Nuclear factor kappa B; FoxO3, Forkhead Box O3; mTOR, mammalian Target of Rapamycin; p70S6K, p70 ribosomal protein S6 kinase; 4E-BP-1, 4E Binding Protein 1; CRP, C-reactive protein; IL-6, Interleukin-6.

### Animal Model

The 7-week-old female athymic nude mice (BALB/c) weighing approximately 25 g were used in this study. The method of orthotopic murine bladder cancer was established as previously described [19]. The mice were anesthetized by using 5 mg ketamine HCl /25g body weight and appropriate measures are taken to minimize pain or discomfort in the animals. The bladder of the anesthetized mice was catheterized through the urethra using a 24-gauge plastic intravenous cannula. To enhance tumor attachment, the bladder was traumatized by instilling 0.1 mL of 0.1 N HCl solution for 15 s followed by neutralization with 0.1 mL of 0.1 N KOH. After HCl and KOH were squeezed from the bladder, the T24 cells (5 × 10^5^ in 100 μL) were instilled through the cannula. After the implantation of cancer cells for 10 days, the mice were divided into 5 weight-matched groups: (1) the normal group; (2) T group (tumor alone group); (3) TGC group (gencitabine + cisplatin treated group): the tumor-bearing mice received gencitabine (1000 mg/m^2^ per 3 days, i.p.) and cisplatin (75 mg/m^2^/week, i.p.); (4) TGCM group (gencitabine + cisplatin + magnolol treated group): the tumor-bearing mice received magnolol (10 mg/kg/day, i.p.) after intraperitoneal injection of gencitabine and cisplatin; and (5) TGM group (gencitabine + magnolol treated group): the tumor-bearing mice received magnolol (10 mg/kg/day, i.p) after intraperitoneal injection of gencitabine (1000 mg/m^2^ per 3 days, i.p.). Each group contained 5 mice. The body weight and health condition of mice were measured and monitored per three days. If any mouse fulfills the criteria for euthanasia established by the Institutional Animal Care and Use Committee (IACUC) such as inappetance, weakness, severe body weight loss, moribund state, and infection that are evaluated by professional veterinarian, the mice will constitute grounds for euthanasia. After 3-week treatment, the mice were sacrificed by using CO_2_, and subsequent tests were performed according to the study design (Fig 1B). The experimental procedures of this study were evaluated and approved by the ethics committee of IACUC of National Defense Medical Center (IACUC-14-044, Taipei, Taiwan).

### Histology and Immunofluorescence

Tissues were fixed with 10% formaldehyde and processed for histopathology, followed by hematoxylin and eosin staining to evaluate the pathological changes in tissues. The intestinal injury was scored according to a modified histological scoring system [20]. For immunofluorescence assay, after the samples were incubated with a specific primary antibody, the fluorescein isothiocyanate-coupled secondary antibody (1:200, Abcam Cambridge, MA, USA) was added for 1 h followed by extensive washing with phosphate-buffered saline tween-20. Subsequently, the targeted proteins were photographed using a fluorescence microscope (Leica, Welzar, Germany). The intensity of immunoreactivity was measured using a densitometer and MetaMorph image analysis software.

### Intestinal Function

The intestinal extracts from jejunum were prepared in 0.9% NaCl supplemented with a proteinase inhibitor. The major intestinal digestive enzyme activities, including those of leucine aminopeptidase (LAP, a digestive enzyme for peptides), lipase (LIP, a digestive enzyme for fats), and amylase (AMYL, a digestive enzyme for sugars), were measured. The biochemical variables were determined using a Fuji DRI-CHEM 3030 analyzer (Fuji Photo Film Co. Ltd., Tokyo, Japan).

### Proteasome Activity

The skeletal muscle (gastrocnemius muscle) samples were dissected and rinsed in ice-cold phosphate-buffered saline to remove blood. The proteasome activity containing chymotrypsin, trypsin, and caspase was determined using a commercially available Proteasome-Glo^™^ 3-Substrate System kit according to manufacturer instructions.

### Western Blotting and Measurement of Muscle Atrophy-Related Regulator

The protein samples (100 μg protein/lane) were loaded and separated on 10% sodium dodecyl sulfate polyacrylamide gel and then transferred to polyvinylidene fluoride membranes and blocked. The membranes were then incubated overnight at 4°C with specific primary antibodies followed by the addition of a horseradish peroxidase-coupled secondary antibody (Abcam, Cambridge, UK). The immunoreactive bands were determined using a chemiluminescence reagent (Amersham International Plc., Buckinghamshire, UK) and were quantified using densitometry and normalized with respective β-actin.

### Statistical Analysis

The data were expressed as mean ± standard error of mean (SEM). The statistical analysis of differences between groups was performed using the one-way analysis of variance with a post hoc Bonferroni test; *P* < 0.05 was considered statistically significant.

## Results

### Magnolol Ameliorates Body Weight Loss

By the end of this study, the untreated tumor-bearing mice (T) had lost 9.6 ± 1.1% of their initial body weight, whereas the normal mice had gained 7.3 ± 0.8% of body weight. The TGC, TGCM, and TGM groups had lost 28 ± 2%, 14.5 ± 1.5%, and 9.5 ± 0.9% of body weight, respectively (Fig 1C). The food intake decreased in the T and all treated groups compared with that in the normal group, and the TGC group exhibited the lowest food intake. Notably, the combined treatment of magnolol groups (TGCM, and TGM) had an increasing trend of the food intake compared with that in the TGC group (Fig 1D). Moreover, the bladder weight, reflecting tumor growth, in various drug-treated groups was markedly reduced compared with that in the tumor-bearing alone group (Fig 1E). Interestingly, the anticancer effect on the TGM group was greater than that in the TGC group. These results indicated that magnolol supplementation not only improved cachexia symptoms but also enhanced the anticancer effect of the chemotherapeutic drugs.

### Magnolol Prevents Enteropathy

**Fig 2:**
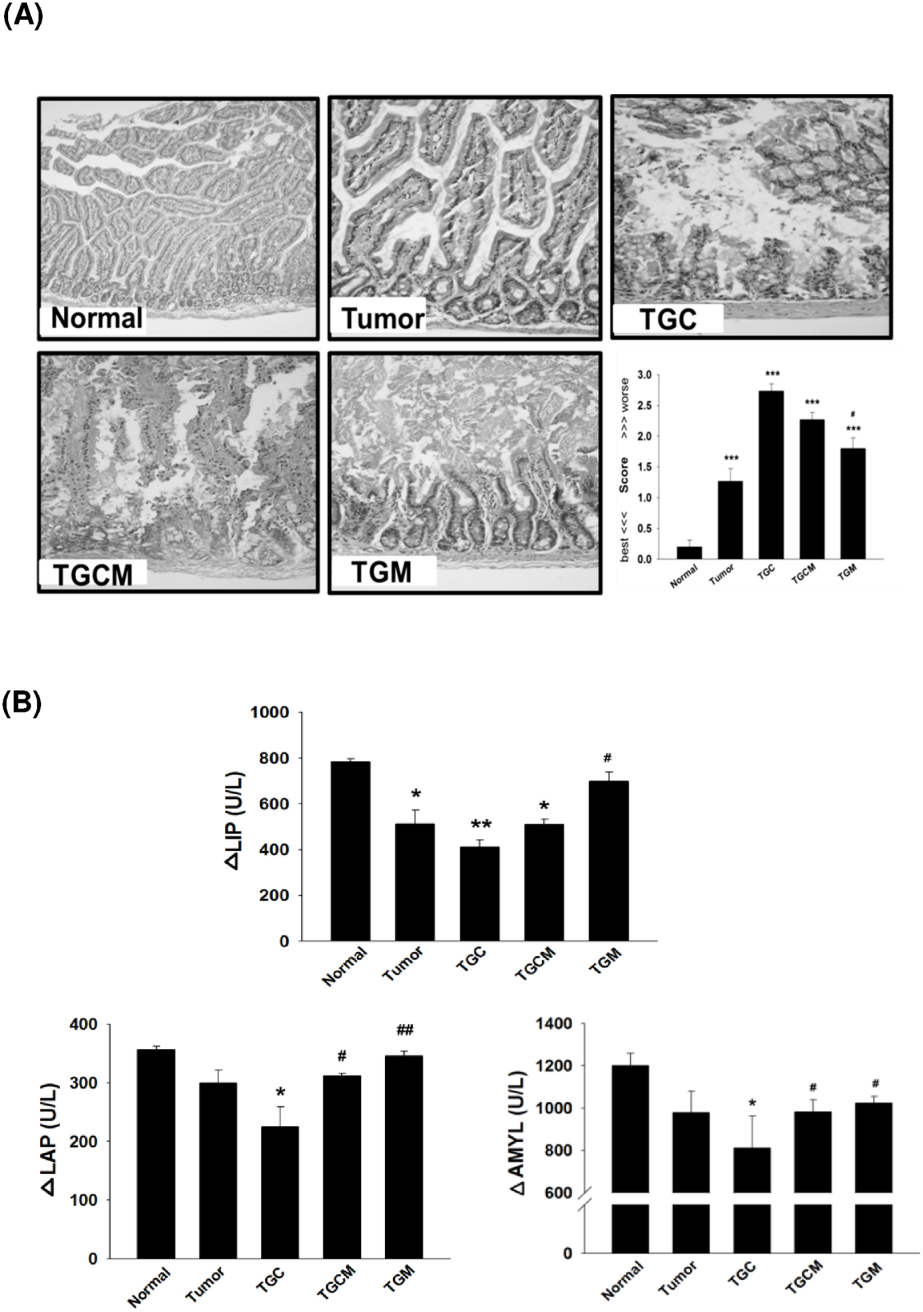
Effects of magnolol on intestinal damage and digestive enzyme dysfunction. The morphological changes in intestinal structure and the grading score were evaluated (A). The intestinal digestive enzyme activity in different groups was determined (B). Data was expressed as mean ± SEM (n = 5). *P < 0.05, **P < 0.01, ***P < 0.001 versus normal group. ^#^ *P* < 0.05, ^##^ *P* < 0.01 versus TGC group.

The enteropathy is a common side effect during chemotherapy, thereby impairing intestinal nutrient absorption and body growth [15]. The histological examinations revealed that the TGC group had intestinal injury the most, whereas the injury was markedly prevented by combined treatment with magnolol (Fig 2A). Furthermore, the decreased intestinal digestive enzyme activities such as LIP, LAP, and AMYL occurring in the TGC group were significantly reversed in TGCM and TGM groups (Fig 2B).

### Magnolol Reduces Muscle Atrophy and Proteasome Activity

**Fig 3:**
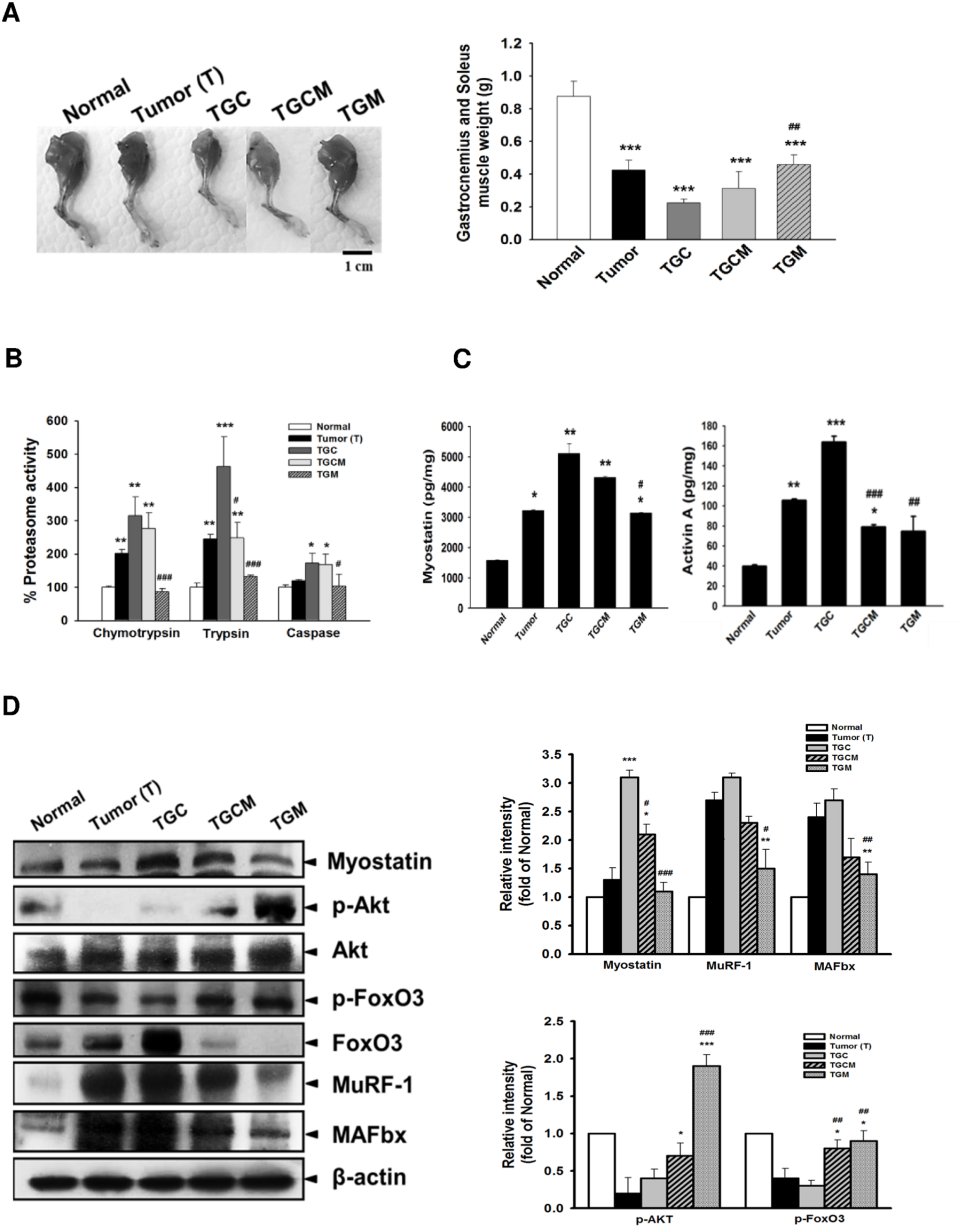
Effects of magnolol on muscle atrophy, proteasome activity and atrogenic gene expression. The images of the muscle of limb and the weight of gastrocnemius and soleus muscle were photographed or measured (A). The proteasome activity (B), the levels of myostatin, and activin A (C), and the protein expression of atrogenic genes (D) in muscle were determined. Data was expressed as mean ± SEM (n = 5). *P < 0.05, **P < 0.01, ***P < 0.001 versus normal group. ^#^ *P* < 0.05, ^##^ *P* < 0.01, ^###^ *P* < 0.001 versus TGC group.

The morphological examination of muscles and the weight of gastrocnemius and soleus muscle clearly indicated that the TGC group lost skeletal muscle mass the most accompanied by the highest proteasome activity among these groups. However, the features observed in the TGC group were greatly attenuated in the TGCM and TGM groups (Fig 3A and 3B). In the TGCM and TGM groups, the protein expression of myostatin, total FoxO3, MuRF 1, and MAFbx in muscle were reduced; conversely, the expression of p-Akt and p-FoxO3 was significantly increased compared with that in the TGC group (Fig 3D). Additionally, the formation of myostatin and Activin A was significantly decreased after combined treatment with magnolol in particular in the TGM group compared with that in the TGC group (Fig 3C).

### Magnolol Attenuates Muscle Atrophy-Related Gene Expression and Increases IGF-1-Regulated Signaling

**Fig 4:**
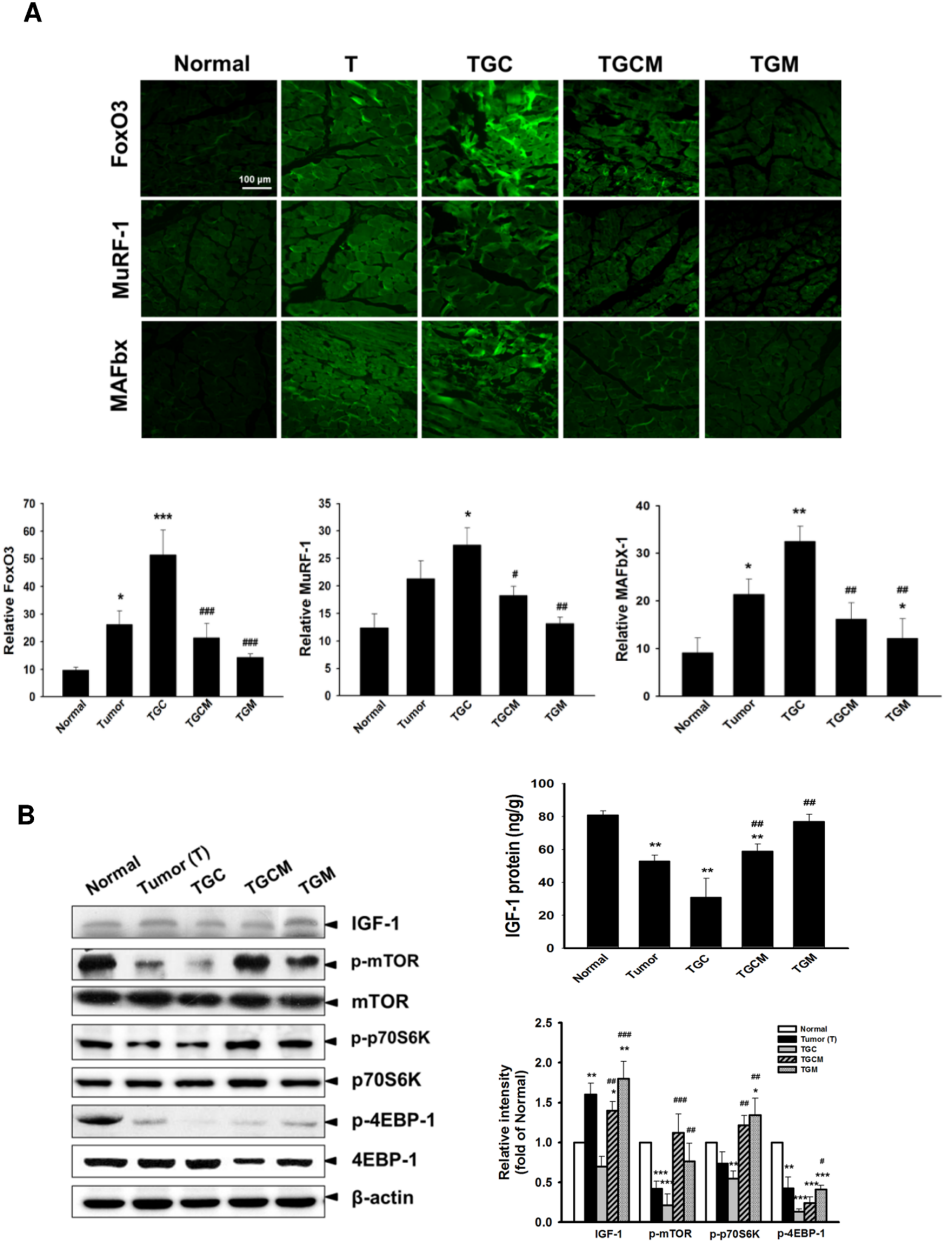
Effects of magnolol on atrogenic gene expression and IGF-1-regulated protein synthesis signaling. The amounts of FoxO3, MuRF-1 and MAFbx determined by immunofluorescence staining (A) and the IGF-1 levels and related protein synthesis signaling pathway in muscle of various groups were determined (B). Data was expressed as mean ± SEM (n = 5). *P < 0.05, **P < 0.01, ***P < 0.001 versus normal group. ^#^ *P* < 0.05, ^##^ *P* < 0.01, ^###^ *P* < 0.001 versus TGC group.

Similarly, the expression of FoxO3, MuRF-1, and MAFbx in muscle determined by immunofluorescence staining was greatly reduced in the TGCM and TGM groups compared with that in the TGC group (Fig 4A). Notably, a marked increase of the production of IGF-1 and the expression of IGF-1, p-mTOR, p-p70S6K and p-4EBP-1 was observed in TGCM and TGM groups compared with that in the TGC group (Fig 4B).

### Magnolol Inhibits Inflammatory Responses

**Fig 5:**
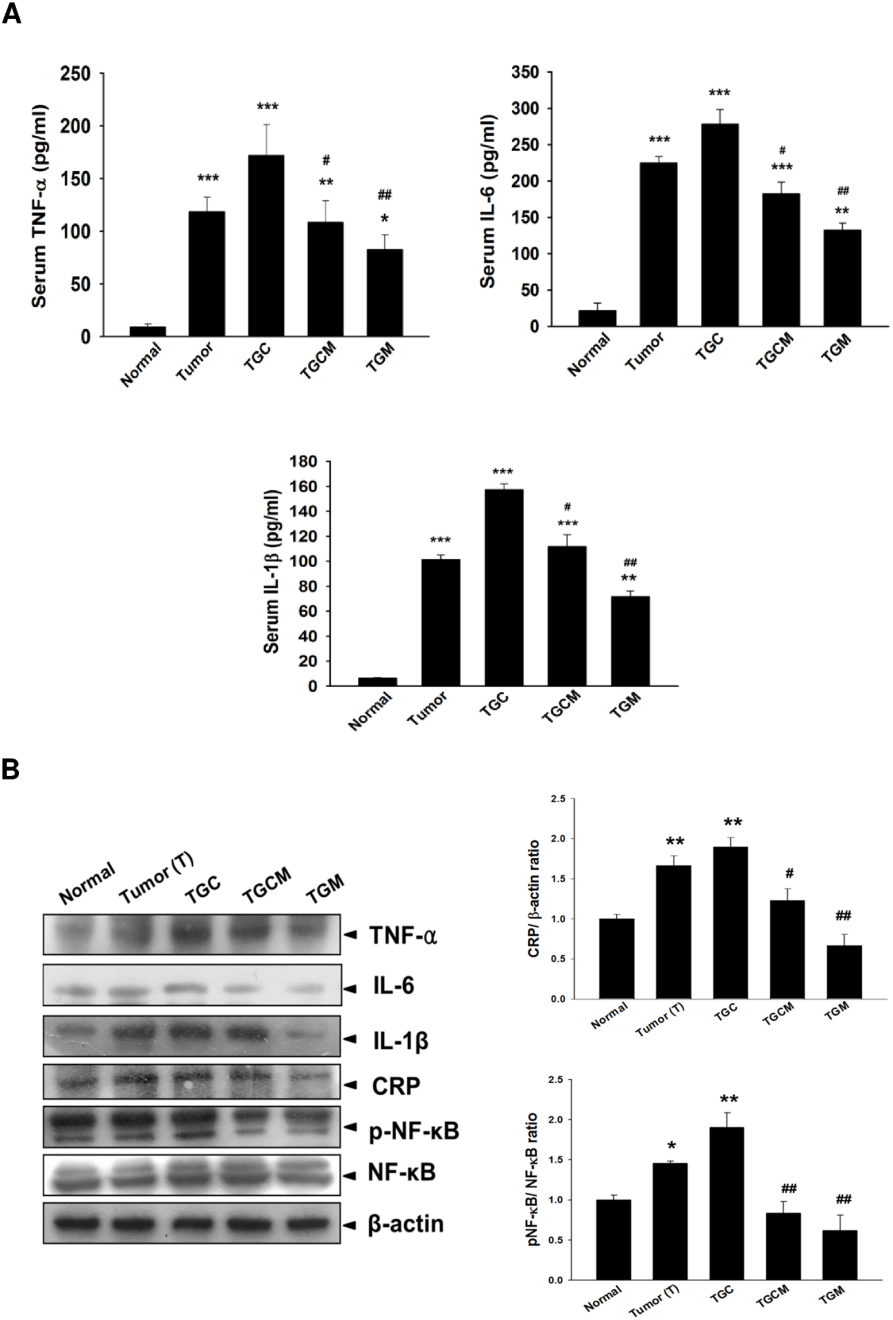
Effects of magnolol on pro-inflammatory cytokine production and NF-κB activation. The serum levels (A) and the protein expression of pro-inflammatory cytokines, CRP and phospho-NF-κB in muscle (B) were measured. Data was expressed as mean ± SEM (n = 5). *P < 0.05, **P < 0.01, ***P < 0.001 versus normal group. ^#^ *P* < 0.05, ^##^ *P* < 0.01 versus TGC group.

The serum levels and muscle expression of proinflammatory cytokines including TNF-α, IL-6, and IL-1β in the TGCM and TGM groups were markedly lower than that in the TGC group (Fig 5A and 5B). In addition, the C-reactive protein (CRP) expression and the NF-κB activation in muscles were significantly inhibited in the TGCM and TGM groups compared with that in the TGC group (Fig 5B).

## Discussion

Epidemiological and clinical studies have confirmed that cancer cachexia is closely associated with poor prognosis and high mortality in cancer patients. Although chemotherapy is a common treatment for cancer, several side effects, including development of cancer cachexia, have been reported. Thus, how to prevent and attenuate chemotherapy-induced cancer cachexia has been a crucial concern during cancer therapy. In this study, we demonstrated that combined treatment with magnolol (TGCM and TGM) effectively alleviates the body weight loss and muscle atrophy occurring in bladder tumor-bearing mice treated with gemcitabine and cisplatin (TGC), thus promoting its clinical use. It is known that maintaining normal intestinal structure and functions is essential for nutritional intake and body growth. Our results revealed that cotreatment with magnolol significantly improved the damage and impaired digestive enzyme activity of the intestinal system in the cachectic animal model, which may enhance the food intake and body weight gain.

The muscle mass is dynamically controlled by the balance between the proteolysis and the synthesis of muscle proteins. Myostatin belonging to the transforming growth factor-β (TGF-β) superfamily is predominantly expressed in skeletal muscles. Myostatin is a critical negative regulator for skeletal muscle growth possibly through inhibition of myoblast proliferation and myogenesis [21]. By contrast, blocking myostatin activity markedly increases the muscle size and physical strength [22]. Activins, a member of the TGF-β superfamily, function as potent inducers for triggering skeletal muscle atrophy. There are two isoforms: Activin A and activin B, and activin A is considered the major form of activins. Interestingly, the actions of myostatin and activins are performed by binding to the same muscle surface receptor complex containing type-II activin receptors (ActRIIA and ActRIIB) and type-I activin receptors (ALK4 and ALK5) [23]. Overproduction of myostatin and activin A has been observed in both cancer patients suffering from cachexia and the animal models of cancer cachexia [24, 25]. Based on our results that the elevated myostatin and activin A levels in muscles of the TGC group were markedly inhibited by magnolol supplementation, magnolol-mediated attenuation of muscle atrophy may be at least in part attributed to suppressing myostatin and activin A release.

Among the isoforms of the FoxO family in skeletal muscles, FoxO3 plays a crucial role in the pathogenesis of muscle wasting. The activity of FoxO is tightly regulated by the change in the subcellular localization of FoxO and its degradation. When FoxO is phosphorylated by Akt, it can be exported from the nucleus in a chaperone 14-3-3-dependent process. The 14-3-3-bound cytoplasmic phosphorylated FoxO proteins are then degraded by the proteasome [26]. Notably, in response to myostatin/activins, the Akt activity is inhibited, thereby resulting in a decrease of FoxO phosphorylation and accumulation of dephospho-FoxO, an active form of FoxO [27]. Then, the activated FoxO translocates into the nucleus, where it activates the transcription of muscle-specific atrogenic genes such as MuRF-1 and MAFbx. Furthermore, FoxO3-regulated autophagy may promote muscle protein degradation [28]. An elevated phosphorylated FoxO3 resulting from activation of Akt and a marked reduction of total FoxO3 protein expression were found in the magnolol combination groups (TGCM and TGM) compared with that in the TGC group. In addition, our unpublished data showed that the association of 14-3-3 with phospho-FoxO3 in the cytoplasm was increased in the TGCM and TGM groups, which may provide a reasonable explanation for enhancing FoxO3 protein degradation. As expected, the FoxO3-mediated downstream MuRF-1 and MAFbx expression and proteasome activity in the muscle tissues were reduced greatly in the TGCM and TGM groups. Collectively, the attenuation of muscle protein breakdown by magnolol may be regulated by suppressing myostatin/activin/FoxO3/MuRF-1/MAFbx signaling pathway and proteasome activity in muscle.

A major role of IGF-1 in stimulating muscle protein synthesis has been accepted [29], which may be modulated by activation of PI3K/Akt/ mammalian target of rapamycin (mTOR) cascade resulting in phosphorylation of protein translational regulators such as p70S6K and 4EBP-1 [30]. Previous study has indicated that the muscle wasting in cancer cachexia was strongly related to downregulation of mTOR/p70S6K/4EBP1 pathway [31]. Conversely, the transgenic mice overexpressing IGF-1 exhibit muscle mass hypertrophy [32]. A novel finding of this study is that a significant decrease of IGF-1 production and expression, as well as the downstream mTOR/p70S6K/4EBP1signaling pathway occurred in the atrophying muscle of the TGC group was markedly reversed in mice of the TGCM and TGM groups. It has been reported that myostatin and proinflammatory cytokines are capable of impairing IGF-1 bioavailability and IGF-1 signaling [33, 34]. Therefore, magnolol-activated IGF-1-dependent processes may be resulted from inhibition of myostatin formation and inflammatory responses. Additionally, IGF-1 is able to trigger Akt-induced FoxO phosphorylation and subsequent degradation [35]. These findings indicate that IGF-1 not only enhances protein synthesis but also prevents muscle protein degradation. Accordingly, induction of protein generation via activation of IGF-1/mTOR/p70S6K/4EBP1 signaling may also contribute to the attenuation of body weight loss by magnolol.

**Fig 6:**
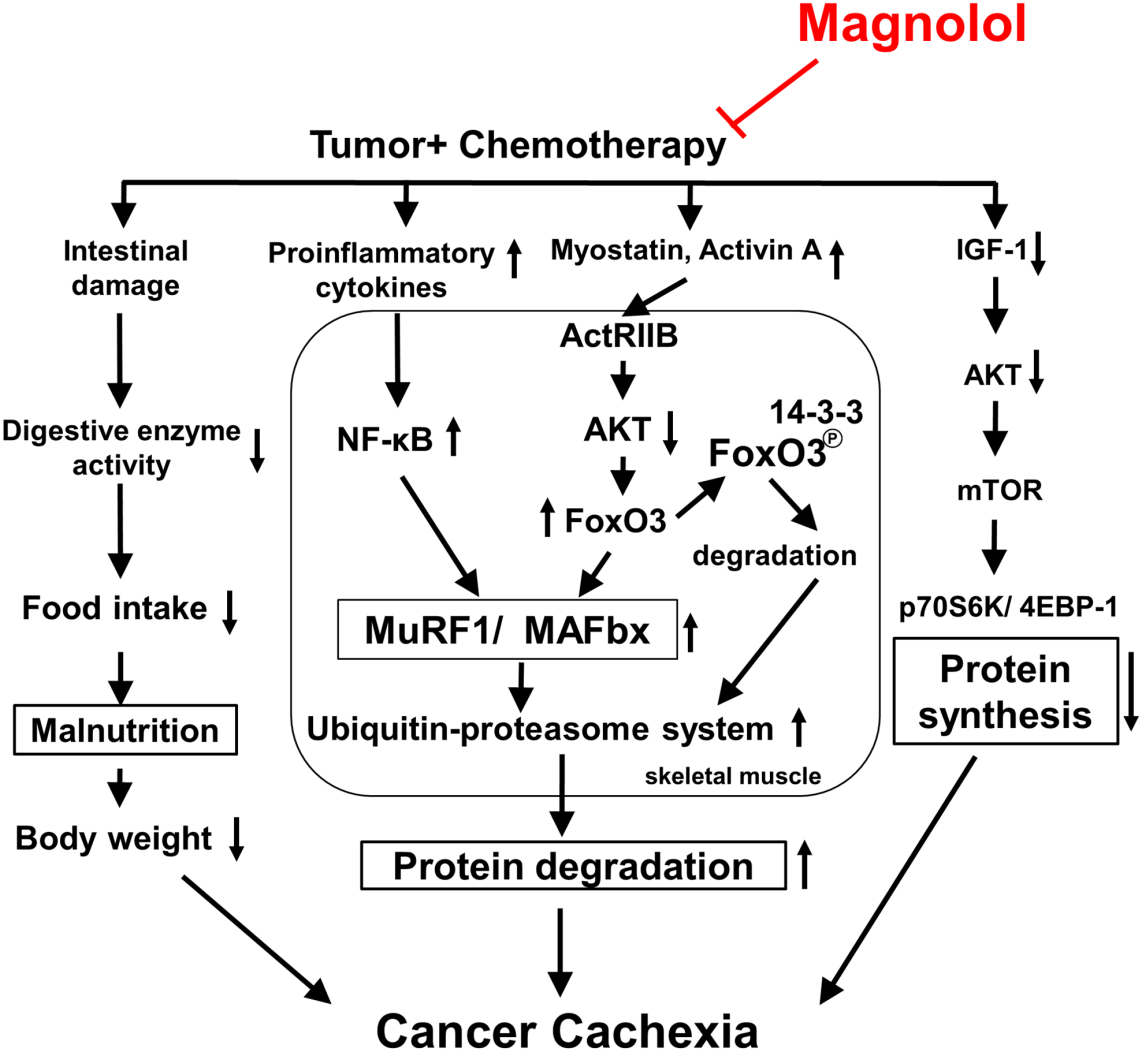
The proposed schematic diagram of signaling pathways for the anti-cachectic activity of magnolol. Combined treatment with magnolol inhibits myostatin/activin/FoxO3 cascade, proinflammatory cytokine formation, and NF-κB activation, leading to suppressing ubiquitin E3 (MAFbx and MuRF1) expression, and proteasome activity, which in turn attenuates the muscle protein proteolysis. Meanwhile, enhancing protein synthesis through activation of IGF-1-regulated signaling, and preventing intestinal damage and anorexia may also contribute to its protective effect. Taken together, magnolol may be a potential supplement for reducing muscle atrophy associated with cancer cachexia during chemotherapy.

The systemic inflammation evoked by NF-κB can induce muscle atrophy through activation of UPS, inhibition of Akt activation, and impairment of muscle differentiation and myogenesis [36]. The proinflammatory cytokines, including TNF-α, IL-6, and IL-1β have been regarded as crucial factors causing cancer cachexia and muscle atrophy [11, 37]. Higher serum levels of proinflammatory cytokines and increased NF-κB activation have been seen in cancer patients with cachexia [38]. Our data showed that magnolol supplementation greatly reduced serum and muscle proinflammatory cytokine levels, NF-κB activation, and CRP, a biomarker of systemic inflammation [39], compared with that in the TGC group, suggesting that the anti-inflammatory effect of magnolol may be involved in its anticachectic activity. Interestingly, we found that the protective effects of the TGM group were generally stronger than that of the TGCM group, supporting that magnolol may be a favorable alternative to replace the more toxic cisplatin for attenuating the toxicity and preventing cancer cachexia development. In conclusion, combined treatment with magnolol markedly reduces chemotherapy-induced cachexia symptoms, particularly body weight loss and muscle atrophy. The underlying molecular mechanisms may include inhibition of myostatin/activin/FoxO3 and NF-κB-mediated muscle protein degradation, and enhancement of IGF-1-dependent protein synthesis (Fig 6). Taken together, magnolol may be a promising chemopreventive agent or supplement to attenuate the skeletal muscle atrophy associated with cancer cachexia.
